# Incidence and outcomes of hospital-associated coronavirus disease 2019 (COVID-19) before and after emergence of the severe acute respiratory coronavirus virus 2 (SARS-CoV-2) omicron variant

**DOI:** 10.1017/ice.2023.29

**Published:** 2023-10

**Authors:** Victoria R. Williams, Charlie Tan, Robert Kozak, Jerome A. Leis

**Affiliations:** 1 Infection Prevention and Control, Sunnybrook Health Sciences Centre, Toronto, Ontario, Canada; 2 Division of Infectious Diseases, Department of Medicine, University of Toronto, Ontario, Canada; 3 Shared Hospital Laboratory, Sunnybrook Health Sciences Centre, Toronto, Ontario, Canada

The severe acute respiratory coronavirus virus 2 (SARS-CoV-2) ο (omicron) variant has been associated with broader community transmission compared to earlier variants but lower mortality.^1,2^ We sought to determine whether similar trends apply to hospital-associated coronavirus disease 2019 (HA–COVID-19) cases.

We conducted a prospective quality improvement study assessing the risk and outcomes of HA–COVID-19 before and after the emergence of the SARS-CoV-2 ο (omicron) variant. From November 1, 2020, to December 14, 2022, all patients admitted to our healthcare facility were tested for SARS-CoV-2 on admission using a reverse-transcriptase polymerase chain reaction (RT-PCR) assay.^3^ Retesting occurred in response to development of new symptoms, after exposure to a SARS-CoV-2–positive roommate, prevalence screening in response to healthcare-associated transmission, and on discharge or transfer to another facility (rehabilitation, long-term care, palliative care and alternative level of care). Each positive test was prospectively assessed to exclude recovered cases and confirm healthcare attribution.

The primary outcome was the incidence of HA–COVID-19 per 1,000 non–COVID-19 patient days defined as all patient days except those while patients were in transmission-based precautions for COVID-19. HA–COVID-19 was defined as positive RT-PCR test or symptom onset ≥5 days after admission, <5 days following transfer from acute care to another facility within our organization, or upon repeat visit within <5 days after discharge. Secondary outcomes were 30-day case fatality rate (CFR) of HA–COVID-19 including only deaths attributable to SARS-CoV-2 infection per the death certificate, admission to an intensive care unit (ICU) for severe COVID-19, and acute-care length of stay from date of detection. Data regarding median age, sex, and attributable service were collected. Incidence and proportion of total COVID-19 cases were compared between the period when the SARS-CoV-2 ο (omicron) variant was dominant (ie, the post-omicron period from December 15, 2021, to December 14, 2022), and the period when earlier variants were circulating (ie, the pre-omicron period from December 15, 2020, to December 14, 2021). Relative risk (RR) with 95% confidence intervals (CIs) were used to compare HA–COVID-19 incidence and CFR between the 2 periods.

Hospital policies were similar between the 2 periods including universal admission testing for SARS-CoV-2, universal masking for all healthcare workers (HCWs), and transmission-based precautions (mask, eye protection, gowns, and gloves) for all patients with suspected or confirmed COVID-19. During the pre-omicron period, N95 respirators were accessible based on point-of-care risk assessment, but during the post-omicron period these became mandatory when providing care for patients with suspected or confirmed COVID-19. COVID-19 vaccine was available for HCWs beginning in December 2020. Visitation restrictions were eased in June 2022, but mandatory masking and syndromic surveillance at point of entry remained in place. We conducted a sensitivity analysis to compare the incidence of HA–COVID-19 before and after this visitation change within the post-omicron period. Data were collected prospectively as part of routine infection prevention and control surveillance, and research ethics review was not required because the study met institutional criteria for exemption as quality improvement research.

Across the 2 periods, 1,866 cases of COVID-19 were identified among acute-care patients: 705 in the pre-omicron period and 1,161 in the post-omicron period. The proportion of total COVID-19 cases that were healthcare-associated was higher during the post-omicron period: 311 (26.8%) of 1,161 versus 34 (4.8%) of 705 (*P* < .01). Similarly, the incidence of HA–COVID-19 increased significantly during the post-omicron period: 1.70 versus 0.19 cases per 1,000 non–COVID-19 patient days (RR, 9.08; 95% CI, 6.37–12.93). Table 1 summarizes patient characteristics and outcomes of patients with HA–COVID-19. During the post-omicron period, 177 (56.9%) of the 311 HA–COVID-19 cases were associated with 23 outbreaks. In comparison, 8 (23.5%) of the 34 cases during the pre-omicron period were associated with 3 outbreaks. Attributable service and proportion identified outside acute care were similar between the 2 periods. The incidence of HA–COVID-19 increased after the visitation policy was revised: 2.12 versus 1.34 cases per 1,000 non–COVID-19 patient days (RR, 1.59; 95% CI, 1.27–1.99). The CFR among HA–COVID-19 cases declined significantly during the post-omicron period: 2.6% versus 14.7% (RR, 0.15; 95% CI, 0.05–0.50). Admission to ICU declined as well but the change was not significant: 3.5% versus 8.8% (RR, 0.38; 95% CI, 0.10–1.43). There was no change in length of stay.

**Table 1.** Incidence, Characteristics, and Outcomes of Hospitalized Patients With Healthcare-Associated COVID-19 During the Pre-omicron and Post-omicron Periods

Characteristic	Pre-omicron Period^a^	Post-omicron Period^b^
Total patient days	187,096	191,172
Non–COVID-19 patient days^c^	181,638	183,007
Hospital admissions	27,648	27,079
Total COVID-19 cases detected, no.	705	1161
Daily COVID-19 case patient census, median (IQR)	5 (2–26)	18 (12–27)
COVID-19 cases from community, no. (%)	671 (95.2)	850 (73.2)
HA–COVID-19 cases, no. (%)	34(4.8)	311(26.8)
Incidence per 1,000 non-COVID-19 patient days	0.19	1.70
Age, median y (IQR)	74.0 (56.5–85.3)	74.0 (62.0–84.0)
Sex, male, no. (%)	22 (64.7)	160 (51.4)
Minimum of 2 doses of COVID-19 vaccine (%)	1(2.9)	279 (89.7)
**Attributable service, no. (%)**		
Medicine	15 (44.1)	150 (48.2)
Oncology	3 (8.8)	53 (17.0)
Surgery	12 (35.3)	78 (25.1)
ICU	1 (2.9)	8 (2.6)
Psychiatry	3 (8.8)	22 (7.1)
Days from admission to detection, median (IQR)	11 (9.0–18.0)	11 (7.0–19.0)
Identified outside acute care, no. (%)	5 (14.7)	44 (14.1)
Outbreak associated, no. (%)	8 (23.5)	177 (56.9)
Identified following roommate exposure, no. (%)	9 (26.5)	69 (22.2)
Treatment for COVID-19, no. (%)^d^	10 (29.4)	112 (36.0)
**Outcomes of patients with HA–COVID-19**		
Length of stay, median d (IQR)	11 (5.0–23.5)	11 (4.0–18.0)
Admitted to ICU, no. (%)	3 (8.8)	11 (3.5)
Case fatality, no. (%)	5 (14.7)	8 (2.6)

Note. HA–COVID-19, healthcare-associated COVID-19; IQR, interquartile range; ICU, intensive care unit.

a December 15, 2020, to December 14, 2021; the period when earlier SARS-CoV-2 variants were circulating.

b December 15, 2021, to December 14, 2022; the period when the SARS-CoV-2 ο (omicron) variant was dominant.

c COVID-19 patient days excluded.

d Treatment includes dexamethasone, remdesivir or ritonavir-nirmatrelvir.

Emergence of the SARS-CoV-2 ο (omicron) variant was associated with a 9-fold higher risk of HA–COVID-19 in our hospital. Using a comparable definition, Klompas et al^4^ reported that the incidence of HA–COVID-19 increased in the first 2 months following the emergence of the SARS-CoV-2 ο (omicron) variant compared to the same 2-month period the year prior.^4^ Holowka et al^5^ compared the SARS-CoV-2 ο (omicron) variant to the SARS-CoV-2 δ (delta) variant only and noted an 8-fold increase in HA–COVID-19 risk. Our study included a longer 12-month comparison that included SARS-CoV-2 wild type, SARS-CoV-2 α (alpha), and SARS-CoV-2 δ (delta) variants combined. In our study, the incidence of HA–COVID-19 was more often associated with outbreaks compared to ancestral variants that were more frequently contained without proceeding to a unit-wide outbreak.

Strategies for responding to the increased risk of HA–COVID-19 in the post-omicron era include uptake of omicron-adapted COVID-19 vaccine boosters for patients and HCWs,^6^ optimizing ventilation in inpatient areas,^7^ and inpatient SARS-CoV-2 testing protocols.^3^ In our study, despite the increased risk of HA–COVID-19, the clinical impact diminished during the post-omicron period, when the CFR of these cases is >80% lower than earlier in the pandemic. The improvement in patient outcome is likely related to viral changes, infection and vaccine-induced immunity, and improvements in therapeutics.^1,8,9^

Our study had several limitations. First, using ≥5 days from admission as the definition for HA–COVID-19 may have included cases that were community acquired, but misclassification was likely uncommon given that the median onset of HA–COVID-19 at our facility was >10 days during both study periods.^10^ In addition, given its shorter median incubation period of <5 days, HA–COVID-19 cases may have been underestimated during the post-omicron period. Second, because patients testing negative for SARS-CoV-2 on admission were not routinely retested within 72 hours, patients incubating virus on admission may have been missed and counted as HA–COVID-19 cases if subsequently detected, although a similar testing strategy was in place during both the pre- and post-omicron periods. Finally, although changes in the visitation policy during the post-omicron period were associated with an increase in HA–COVID-19, this does not account for the overall 9-fold increase in risk that we observed.

The SARS-CoV-2 ο (omicron) variant poses a higher risk of HA–COVID-19 compared to other variants although patient outcomes have improved.
